# Bioengineering scalable and drug-responsive in vitro human multicellular non-alcoholic fatty liver disease microtissues encapsulated in the liver extracellular matrix-derived hydrogel

**DOI:** 10.17179/excli2023-6878

**Published:** 2024-03-25

**Authors:** Negar Asadollahi, Mohammad Amin Hajari, Mahmoud Alipour Choshali, Mohammad Ajoudanian, Seyed Ali Ziai, Massoud Vosough, Abbas Piryaei

**Affiliations:** 1 Department of Regenerative Medicine, Cell Science Research Center, Royan Institute for Stem Cell Biology and Technology, ACECR, Tehran, Iran; 2 Department of Developmental Biology, University of Science and Culture, Tehran, Iran; 3 Department of Cell Engineering, Cell Science Research Center, Royan Institute for Stem Cell Biology and Technology, ACECR, Tehran, Iran; 4 Department of Stem Cells and Developmental Biology, Cell Science Research Center, Royan Institute for Stem Cell Biology and Technology, ACECR, Tehran, Iran; 5 Department of Medical Biotechnology, School of Advanced Technologies in Medicine, Shahid Beheshti University of Medical Sciences, Tehran, Iran; 6 Department of Pharmacology, School of Medicine, Shahid Beheshti University of Medical Sciences, Tehran, Iran; 7 Experimental Cancer Medicine, Institution for Laboratory Medicine, Karolinska Institute, Huddinge, Sweden; 8 Department of Biology and Anatomical Sciences, School of Medicine, Shahid Beheshti University of Medical Sciences, Tehran, Iran

**Keywords:** non-alcoholic fatty liver disease, metabolic dysfunction-associated fatty liver disease, liver microtissue, bioengineering, drug screening, liraglutide

## Abstract

Non-alcoholic fatty liver disease (NAFLD) is a high-prevalence and progressive disorder. Due to lack of reliable *in vitro* models to recapitulate the consecutive phases, the exact pathogenesis mechanism of this disease and approved therapeutic medications have not been revealed yet. It has been proven that the interplay between multiple hepatic cell types and liver extracellular matrix (ECM) are critical in NAFLD initiation and progression. Herein, a liver microtissue (LMT) consisting of Huh-7, THP-1, and LX-2 cell lines and human umbilical vein endothelial cells (HUVEC), which could be substituted for the main hepatic cells (hepatocyte, Kupffer, stellate, and sinusoidal endothelium, respectively), encapsulated in liver derived ECM-Alginate composite, was bioengineered. When the microtissues were treated with free fatty acids (FFAs) including Oleic acid (6.6×10^−4^M) and Palmitic acid (3.3×10^−4^M), they displayed the key features of NAFLD, including similar pattern of transcripts for genes involved in lipid metabolism, inflammation, insulin-resistance, and fibrosis, as well as pro-inflammatory and pro-fibrotic cytokines' secretions and intracellular lipid accumulation. Continuing FFAs supplementation, we demonstrated that the NAFLD phenomenon was established on day 3 and progressed to the initial fibrosis stage by day 8. Furthermore, this model was stable until day 12 post FFAs withdrawal on day 3. Moreover, administration of an anti-steatotic drug candidate, Liraglutide (15 μM), on the NAFLD microtissues significantly ameliorated the NAFLD phenomenon. Overall, we bioengineered a drug-responsive, cost-benefit liver microtissues which can simulate the initiation and progression of NAFLD. It is expected that this platform could potentially be used for studying molecular pathogenesis of NAFLD and high-throughput drug screening.

See also the graphical abstract(Fig. 1).

## Introduction

Non-alcoholic fatty liver disease (NAFLD) more recently also referred to as metabolic dysfunction-associated fatty liver disease (MAFLD) is the prevailing chronic liver disease, with a prevalence of 30 percent of population (Younossi et al., 2023[51], 2016[52]). Since the fatty liver may coincide with the consumption of alcohol in non-symptomatic person or patients suffering from viral hepatitis, obesity, and particularly some metabolic disorders, including prediabetes and diabetes mellitus, the term MAFLD accurately reflects the pathophysiology of the disease (Eslam et al., 2020[16][17]; Evans, 2002[18]; Streba et al., 2015[46]). 

The diagnosis of NAFLD requires the presence of at least five percent steatosis in the absence of any other cause of liver disease, particularly highlighting alcohol consumption (Ludwig et al., 1980[34]). However, the diagnosis criteria for MAFLD utilize the same standard for hepatic steatosis but also require the presence of at least one metabolic dys-regulatory factor (Eslam et al., 2020[17]; Evans, 2002[18]). The main reason for altering the terminology was to emphasize the significance of metabolic abnormalities. Although, the concept of MAFLD has been accepted by many specialists considering it to more precise represents relevant risk factors compared to the term NAFLD, the new nomenclature has not yet gained widespread usage (Younossi et al., 2021[53]). It appears that altering the nomenclature is appropriate when new knowledge about the disease's molecular basis, significant changes in risk assessment, or other fundamental aspects of the disease are identified (Fouad et al., 2021[21]; Younossi et al., 2021[53]).

The main cause of this disease is an imbalance in diverse metabolism processes, such as lipolysis, synthesis of triglycerides, secretion of very low-density lipoproteins, and *de novo* lipogenesis and dietary factors that lead to the excessive accumulation of lipids in the form of triglycerides within the liver (Alwahsh and Gebhardt, 2017[2]; Gluchowski et al., 2017[24]). NAFLD encompasses a wide spectrum of liver diseases, from simple steatosis to non-alcoholic steatohepatitis (NASH), as well as liver fibrosis and cirrhosis. Steatosis is characterized by lipids accumulating to more than 5 % of total liver weight without signs of hepatocellular injury (Chalasani et al., 2018[8]). 

Following lipid accumulation in the hepatocytes, Kupffer cells (KCs) are activated, and the disease progresses to NASH. Reciprocal interactions between the Kupffer cells, hepatocytes, and liver sinusoidal endothelial cells (LSECs) lead to the release of different cytokines, including platelet-derived growth factor (PDGF), transforming growth factor-β1 (TGF-β1), and vascular endothelial growth factor (VEGF). These cytokines cause hepatic stellate cells (HSCs) to activate and convert into myofibroblasts that produce fibrosis-associated proteins, such as collagen and fibronectin, progressing the disease to fibrosis and then cirrhosis (Crespo Yanguas et al., 2016[12]; Kazankov et al., 2019[30]; Tsuchida and Friedman, 2017[49]). 

Despite the high prevalence of NAFLD, there is no approved drug for its therapy (Friedman et al., 2018[22]). Therefore, carrying out preclinical studies are crucial to enhance our knowledge of the disease's pathophysiology so as to develop effective medicine for it. There are different NAFLD models already, including animal models and some *in vitro* platforms. However, the animal models fail to completely capture human NAFLD pathophysiology and histopathology (Even et al., 2017[19]; Ma et al., 2021[35]). In this regard, it has been known that some candidate medicines which are effective on animals do not show efficacy on humans due to differences in genetics, metabolism condition, insulin resistance, and fibrosis mechanisms (Munteanu et al., 2016[37]). 

It is expected that a suitable *in vitro* human NAFLD model, due to the use of human cells, could address the drawbacks of the animal models. For this purpose, during the past decades, a variety of *in vitro* models of NAFLD with different complexity levels, from two-dimensional (2D) monoculture cells to more advanced three-dimensional (3D) co-culture systems, have been developed (Barbero-Becerra et al., 2015[5]; Feaver et al., 2016[20]; Graffmann et al., 2016[27]; Zahmatkesh et al., 2022[55]). The monoculture platforms, traditionally created using primary human hepatocytes (PHH), lack the important roles of non-parenchymal cells in the pathogenesis of NAFLD (Chen et al., 2018[10]). NAFLD implies a complex interaction between different types of cells all of which must be considered in the development of an *in vitro* model. Therefore, incorporating the non-parenchymal cells (NPCs including KCs, HSCs, and LESCs) with the liver parenchymal cells (hepatocytes) should be done to recapitulate NAFLD's initiation and progression because each cell type has a specific role in steatosis, inflammation, and fibrosis (Cho et al., 2021[11]). 

In addition to the NPCs, biophysical and biochemical properties of the extracellular matrix (ECM) are parameters that contribute greatly to normal physiology as well as the pathogenesis of diseases (Poole and Arteel, 2016[43]; Sonbol, 2018[45]). Recent studies have shown that during the progress of liver fibrosis, dynamic changes in the matrix's stiffness have a critical role in activating the hepatic stellate cells (Godoy et al., 2013[25], Wells, 2005[50]). In addition, it has been observed that ECM-associated bioactive molecules, such as chemokines, growth factors, and damage-associated molecular patterns (DAMPs), are critical regulators during liver fibrosis (McQuitty et al., 2020[36]; Ortiz et al., 2021[40]).

Overall, it has been proven that a combination of parenchymal cells and major NPCs together with a proper ECM are necessary to generate an *in vitro* biomimetic tissue equivalent construct. More recently, in the field of liver tissue engineering and NAFLD modeling, some interesting attempts have been done. In this regard, Feaver and colleagues developed a transwell co-culture condition in which PHHs were seeded at the bottom of the transwell whereas human HSCs and KCs were cultured on the top of the transwell. The co-culture was implemented to a microfluidic system and nourished by culture media containing increasing levels of glucose, FFA, and insulin over 10 days to induce NAFLD. This *in vitro* model was comparable with the NASH liver in some indices, including inflammation, increased oxidative stress, mitochondrial dysfunction, lipid accumulation, and fibrosis, although in the co-culture system, the PHHs have no physical interactions with the NPCs (Feaver et al., 2016[20]). Through co-culturing, HepG2, KCs, and HUVECs in gelatin methacryloyl (GelMA), Suurmond and colleagues fabricated a hepatic spheroid to evaluate advancement of steatosis to NASH. They found that KCs could be induced to produce pro-inflammatory cytokines, and also higher levels of cellular stress happened upon exposing the liver spheroid to free fatty acids (FFAs) (Suurmond et al., 2019[48]).

By co-differentiating of human pluripotent stem cells (hPSCs) into liver parenchymal and stromal cells and incorporating them in Matrigel, Ouchi and colleagues generated a human liver organoid. The transcriptome of this interesting organoid, which included hepatocyte-like, stellate-like, and Kupffer-like cells, has good similarities to normal liver tissues. They demonstrated gradual rise in intracellular lipids accumulation during exposure of the liver organoids to the increasing doses of FFAs. This organoid demonstrates the stepwise initiation and progression of steatohepatitis and produces some inflammatory cytokines including interleukins-6 and 8 (IL-6, 8), and tumor necrosis factor-α (TNF-α). Moreover, prolonged supplementation of FFAs showed hepatocyte ballooning and upregulation of vimentin and α-smooth muscle actin (α-SMA) transcripts in the liver organoids (Ouchi et al., 2019[41]). 

Taken together, there are some major obstacles in almost all of the previously developed models to make them applicable, including the availability and recruitment of all contributing cells or using the appropriate ECM. Moreover, in the pipeline for high throughput production and commercialization, high costs as well as the lack of reproducibility and scalability are the other bottlenecks that have to be overcome (Ouchi et al., 2019[41]; Ströbel et al., 2021[47]; Suurmond et al., 2019[48]).

In the present study, to achieve affordable, reproducible, and scalable NAFLD model, we bioengineered a multicellular liver microtissue (LMT), composed of available human cell lines (Huh-7, THP-1, and LX-2) and human umbilical vein endothelial cells (HUVEC) which can be a proper substitute for the four main liver cells (hepatocyte, Kupffer, stellate cells, and sinusoidal endothelium, respectively), encapsulated in liver-derived ECM. Exposing the LMTs to FFAs recapitulates the hallmarks of NAFLD initiation and progression, such as lipid accumulation, inflammation, and fibrosis. Moreover, responsiveness to an anti-steatosis drug candidate, Liraglutide, was demonstrated in the bioengineered model.

## Materials and Methods

### Ethical statement 

All the experiment protocols of this project were in consent with the guidelines of the Ethics Committee of Shahid Beheshti University of Medical Sciences (IR.SBMU.MSP.REC.1400.305). In this regard, for isolating primary HUVEC from the umbilical cords, informed consent was obtained from the parents before the birth of healthy term neonates. All materials were purchased from Gibco (Gaithersburg, MD, USA) unless stated otherwise.

### Cell culture

The three cell lines were used in the present study: Huh-7 (RRID:CVCL_0336), THP-1 (RRID:CVCL_0006), and LX-2 cells (RRID:CVCL_5792) were obtained from the Royan Stem Cell Bank (Iran) and HUVECs were extracted from the umbilical cord of healthy newborns. Huh-7 and LX-2 cells were cultured in high-glucose Dulbecco's Modified Eagle Medium (HG-DMEM, 11995-040). THP-1 cells were expanded in Roswell Park Memorial Institute (RPMI) medium (31800-105). HUVECs were isolated from the umbilical vein using 1 % collagenase (17104-019) and expanded in Royan Endothelial Growth Medium (REGM, Royan Institute, Tehran, Iran) up to passage 4. All culture media were supplemented with Fetal Bovine Serum (FBS, 16140-071, 2 % for LX-2, and 10 % for others), 1 % penicillin/streptomycin (Pen/Strep, 15070-063), 1 % non-essential amino acids (11140-035), 1 % GlutaMax (35050-038), and 0.1 mM β-mercaptoethanol (M7522, Sigma-Aldrich, St Louis, MO, USA). 

### Hydrogel preparation 

Liver-derived ECM hydrogel (LEMgel) was produced according to our previously reported protocols with a few modifications (Saheli et al., 2018[44]). Briefly, sheep liver was frozen and cut into 2 mm-thick pieces and decellularized using 1 % sodium dodecyl sulfate (SDS, 436143, Sigma-Aldrich, St Louis, MO, USA) and 1 % Triton X100 (108643, Merck, Burlington, MA, USA). Then, the decellularized tissue was lyophilized using a Christ lyophilizer (Alpha 1-2 LDplus, Osterode am Harz, Germany), ground and sterilized, using UV irradiation, digested enzymatically using 1 % pepsin (P6887, Merck, Burlington, MA, USA) in 0.5 M acetic acid (Sigma-Aldrich, St. Louis, MO, USA), and stirred at 4 °C for 48 h to prepare a stock solution of 6 mg/ml LEMgel. Alginate (Alginic acid sodium salt, Sigma-Aldrich, 180,947) was dissolved in distilled water to produce a 2 % (W/V) solution. Next, the LEMgel was neutralized (pH 7.4) and mixed with the alginate solution at an equal ratio to achieve a homogenous mixture of the composite with a final concentration of 3 mg/ml LEMgel and 1 % alginate.

### Microtissue generation

**Figure 2:**
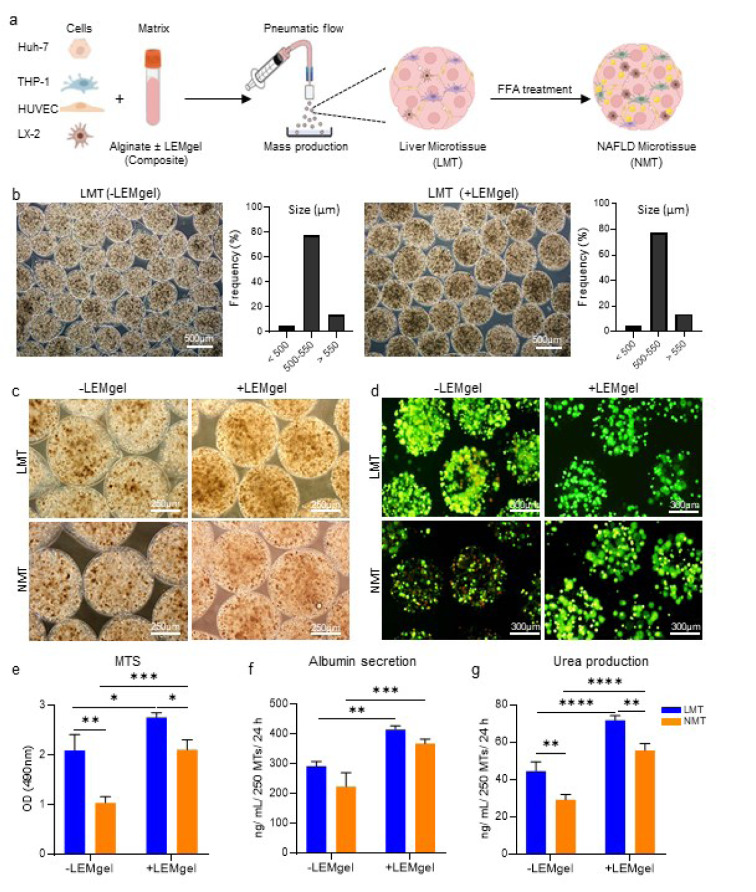
Development and characterization of the liver microtissue (LMT) and induced NAFLD microtissue (NMT) in the absence or presence of LEMgel (-LEMgel and +LEMgel groups, respectively). a) Schematic display of the LMT generation and NAFLD induction. b) Phase-contrast micrographs on day 1 showed homogeneous distribution of cells inside the -/+LEMgel liver microtissues structure. The graphs represent the size distribution of the generated microtissues (n = 100). c) Phase-contrast and d) fluorescence micrographs representing morphology and cell viability, demonstrated by live/dead (green/red) staining, for the LMT and the NMT induced through 8 days incubation of the microtissues with FFA. e) Quantification of cell viability using MTS assay (n = 3), and f and g) the microtissues functionality, considering albumin secretion and urea production on day 8 (n = 3). Data are shown as mean *± *SD. *p < 0.05, **p < 0.01, ***p < 0.001, and ****p < 0.0001.

According to Darakhshan et al., the generation of LMTs was performed using the extrusion method adopted in our laboratory (Darakhshan et al., 2020[13]). A suspension of the four cell types, including Huh-7, THP-1, HUVEC, and LX-2, at a 7:2:2:1 ratio was prepared and centrifuged. Next, the cells were gently mixed to the composite or 1 % alginate to make a homogeneous cell suspension at a concentration of 7×10^6^ cells/ml for generating +LEM or -LEM microtissues, respectively. Then, the resulting cell-laden hydrogel was loaded into a syringe pump (Harvard Apparatus, Holliston, MA, USA, PHD ULTRA^TM^) to produce the LMTs in diameter at 500 μM (Figure 2a(Fig. 2)). Finally, the produced LMTs were rinsed using DMEM and maintained in a co-culture medium consisting of HG-DMEM, RPMI, and REGM based on the cell ratio in the LMTs, supplemented with 1 % FBS, 1 % fatty acid-free Bovine Albumin Serum (FAF-BSA, A8806, Sigma-Aldrich, St. Louis, MO, USA), 1 % Pen/Strep, 1 % L-glutamine, and 1 % non-essential amino acids. The size distribution of the generated LMTs for each group was measured on day 1 using DP2-BSW software linked with a phase-contrast microscope (Olympus, IX71, Tokyo, Japan).

### NAFLD induction

Free fatty acids composed of Oleic Acid and Palmitic Acid (Sigma-Aldrich, O1383, and P0500, respectively) were used for NAFLD microtissue (NMT) induction. Briefly, the FFAs were dissolved in 0.1 M NaOH and incubated at 70 ºC for 10-15 min until the solution was clear. FAF-BSA was dissolved in 0.9 % NaCl and stirred at 37 ℃. Next, the clear solution of FFAs was added to the FAF-BSA solution and then added to the co-culture media at a working concentration of 6.6×10^−4^ M Oleic Acid, 3.3×10^−4^ M Palmitic Acid, and 1 % FAF-BSA (Lasli et al., 2019[32]). The co-culture media was supplemented with 1 % FBS to avoid changes in the FFAs:BSA ratio by binding the serum albumin to the FFAs; the medium was refreshed every other day (Suurmond et al., 2019[48]).

### Evaluating the stability of the NAFLD microtissues

To assess whether the NAFLD state is stable in the resultant NMT or would revert to a healthy condition, after 3 days of exposure to FFAs, the FFAs were withdrawn from the culture media up to day 12. Then, the amount of intracellular lipid accumulation was evaluated using Nile red staining. 

### Assessment of the NAFLD microtissue drug responsivity

To evaluate the drug responsivity of the NMT, after NAFLD establishment in the initial 3 days, the NMT was treated with different doses (15, 20, and 25 μM) of Liraglutide, from day 4 to 12. Next, using Nile red staining and gene expression, the drug responsivity of the resultant NMT was evaluated.

### Cell viability assessment 

Cell viability was assessed using live/dead Viability/Cytotoxicity Kit (L3224, Invitrogen Carlsbad, CA, USA) and 3-(4,5-dimethylthiazol-2-yl)-5-(3-carboxymethoxyphenyl)-2-(4-sulfophenyl)-2H-tetrazolium) (MTS) assay. For the live/dead assay, on the target time point, the microtissues were washed by PBS and stained using 2 µM calcein-AM and 4 µM ethidium homodimer at room temperature for 30 min. Next, the micrographs were obtained using an inverted fluorescence microscope (IX71, Olympus, Tokyo, Japan). To prepare the MTS assay, on the appropriate time point, 100 LMTs or NMTs were incubated in one well of 96-well containing a final volume of 100 µl co-culture media. Then, 20 µl of the MTS solution was added to each well and incubated at 37 °C for 3 hours. Finally, the absorbance was recorded at 490 nm using a microplate reader (51118650, Thermo Scientific, Multiskan Spectrum). 

### Gene expression analysis

Transcript assessments were performed for 1444000 microtissues on each time point for all groups. Total RNA was extracted using RNeasy Micro Kit (74004, QIAGEN, Hilden, Germany) according to the manufacturer's protocol. Next, 1 µg of RNA was utilized as a template to synthesize cDNA using human specific primers (Supplementary Table 1) and a PrimeScript™ Reverse Transcriptase Kit (Takara Bio, Inc., Kusatsu-Shi, Japan) according to the manufacturer's guidelines. The amounts of transcripts of the target genes were normalized to an internal control, the glyceraldehyde-3-phosphate dehydrogenase (GAPDH), and calibrated against the expression values of the related LMT. The comparative CT method (2^−∆∆Ct^) was used to analyze and present the data.

### Hepatic-related protein and inflammatory cytokines secretion, and urea production assays

To evaluate the functionality of the generated microtissues, secretion of albumin (ALB), IL-6, TGF-β, and amino-terminal pro-peptide for type III procollagen (P3NP) were assessed using Enzyme-Linked Immunosorbent Assay (ELISA) commercial kits (ELH-ALB, Raybiotech, Peachtree Corners, GA, USA; D6050, R&D System, Minneapolis, MN, USA; DY240-05, DuoSet ELISA kit, R&D Systems, Minneapolis, MN, USA; MBS455322, MyBioSource, San Diego, California, USA, respectively) according to the manufacturer's protocol. Furthermore, the amount of urea production in the microtissue secretome was measured using a Colorimetric Assay kit (MBS2601488, MyBioSource, San Diego, California, USA) based on the manufacturer's guideline. For this purpose, in all cases, 250 microtissues were cultured in each well of a 24-well plate, and the supernatants were collected after 24 hours of incubation. 

### Intracellular lipid accumulation analysis

Using Oil red (O0625, Sigma-Aldrich, St. Louis, MO, USA) and Nile red (19123, Sigma-Aldrich, St. Louis, MO, USA), staining intracellular lipid accumulation was evaluated. Oil red is a lipid-soluble dye that stains neutral triglycerides and lipids red. Nile red is a lipophilic dye that can intensively be fluorescent in a lipid-rich environment, with different colors ranging from deep red for membrane phospholipids to orange-yellow gold for accumulated triglycerides (Diaz et al., 2008[14]). For this process, some microtissues were transferred to a 24-well plate at the appropriate times, washed twice by PBS, and fixed using 4 % paraformaldehyde (at 4 ℃, overnight). For Oil red staining, the microtissues were incubated in 60 % isopropanol for 5 minutes and then in 0.3 % Oil red solution for 10-20 minutes. Next, the microtissues were visualized and micrographs were taken using a light microscope. To quantify lipid accumulation, the microtissues were incubated in 10 µM Nile red in methanol at room temperature for 10 min. After washing via PBS, appropriate fluorescent micrographs were acquired from the microtissues using an IX71 Olympus microscope. Finally, the mean fluorescent intensity of the accumulated neutral lipids was quantified for at least 30 microtissues per group using the ImageJ software (Version 1.2.4, RRID:SCR_003070).

### Statistical analysis

All data are presented as means ± standard deviation (SD). The data were analyzed using two-tailed Student's t-test to compare the two groups, and in order to compare more than two groups, one-way or two-way ANOVA were used. P-values < 0.05 were considered statistically significant. The data were analyzed using Prism software (V8.4.3, RRID:SCR_002798).

## Results

### Development and characterization of LMT and NMT

To provide an appropriate matrix for liver microtissue generation, we used a biomimetic composite composed of LEMgel and alginate. Liver decellularization and LEMgel production was prepared according to our previous study (Saheli et al., 2018[44]), which assured that the main components of liver ECM were preserved during the process (Supplementary Figure 1). Moreover, in order to enable mass production of the microtissues by an air-driven droplet generator system, we added 1 % alginate to the LEMgel to allow rapid gelation of the resultant composite and to stabilize the microtissues (Darakhshan et al., 2020[13]). On the other hand, to investigate the impact of LEMgel on the generated microtissues, a mixture of Huh-7, THP-1, HUVEC, and LX-2 cells (Supplementary Figure 2) at a ratio of 7:2:2:1 was embedded in the composite or alginate to achieve the +LEM or -LEM microtissues, respectively (Figure 2a(Fig. 2)).

Both of the ±LEMgel LMTs showed a homogenous morphology and cell distribution; more than 75 % of them have a diameter of 500 μm 24 hours after generation (Figure 2b and c(Fig. 2)). Comparing the microtissues on day 8, a higher number of dead cells were observed in the -LEMgel groups (Figure 2d(Fig. 2) and Supplementary Figure 3). Furthermore, considering cell viability, it was found that the presence of LEMgel significantly increased cell preservation and cell viability was dramatically lower in NMT compared to the LMT (Figure 2e(Fig. 2)). Although no significant change in albumin secretion was observed by comparing LMT and NMT, a considerably higher albumin presence was detected in the secretome of the +LEMgel microtissues in comparison to their -LEMgel counterparts (Figure 2f(Fig. 2)). In the case of urea production, the amount for +LEMgel groups was significantly higher compared to those of -LEMgel ones and also for the LMT groups in comparison to their relevant NMT microtissues (Figure 2g(Fig. 2)).

Taken together, these results indicate that the presence of LEMgel is impactful on cell viability and the functionality of generated LMT and NMT. Therefore, we decided to conduct the rest of our experiments using +LEMgel microtissues and concentrate on the main goal of the study, i.e. the *in vitro* NAFLD model.

### Complementary characterization of NMT on day 8

**Figure 3:**
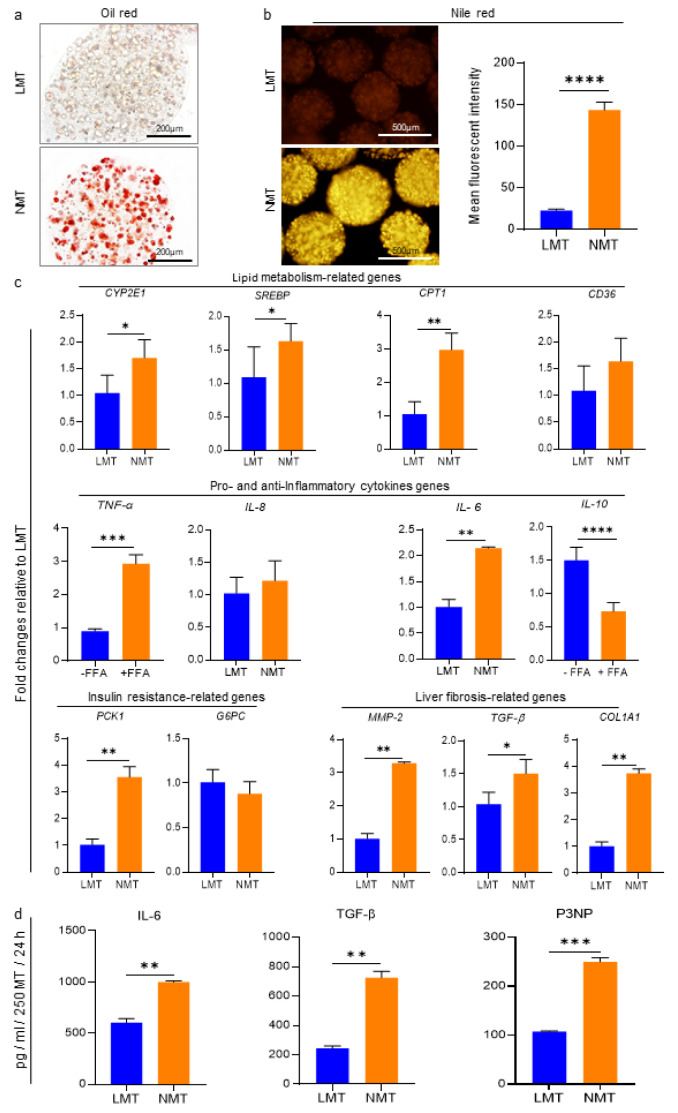
Complementary evaluations of the induced NAFLD microtissue (NMT) generated in the presence of LEMgel compared to the control liver microtissue (LMT) on day 8. a) Representative phase-contrast micrographs from Oil red stained microtissues demonstrate intracellular lipid accumulations, particularly in the NMT. b) Fluorescence micrographs and quantification of accumulated lipids, using Nile red staining and calculating the mean fluorescence intensity, showed significantly more lipid storage in the NMT compared to the LMT (n = 6). c) Gene expression of NMT, compared to the control LMT, on day 8. The alterations in evaluated gene expressions involved in lipid metabolism, pro- and anti-inflammatory cytokines, insulin resistance, and liver fibrosis confirmed that the generated NAFLD mode was successfully established in the NMT on the 8^th^ day through exposure to FFAs. Data were normalized against *GAPDH *and are presented as fold changes calibrated to expression values of LMT on day 8 (n = 3). d) Quantification of secreted IL-6, TGF-β, and P3NP in the microtissues supernatant using ELISA on day 8 (n = 3). The results are represented as mean ± SD. *p < 0.05, **p < 0.01, ***p < 0.001, and ****p < 0.0001.

NAFLD is typically identified by lipid accumulation in hepatic cells; therefore, Oil red and Nile red staining were prepared on day 8 to prove the NAFLD model. The micrographs taken from both staining methods demonstrated that there are more lipid droplets in the NMT compared to the LMT. Furthermore, quantifying the mean fluorescent intensity for Nile red staining displayed significantly higher levels of lipid accumulation in NMT (Figure 3a, b(Fig. 3)). 

To characterize the NAFLD phenotype at the molecular level, gene sets for the hallmarks of NAFLD, such as lipid metabolism, inflammatory response, insulin resistance, and collagen synthesis, were evaluated (Figure 3c(Fig. 3)). The results indicated that transcripts for genes related to lipid metabolism, including cytochrome P450 2E1 (CYP2E1), sterol regulatory element-binding protein (SREBP), and carnitine palmitoyltransferase 1 (*CPT1*)**,** were significantly higher in NMT compared to LMT. In comparison to LMT, the gene expression for pro-inflammatory cytokines, such as TNF-α and IL-6 were, significantly upregulated in NMT, while the transcripts for anti-inflammatory cytokine IL-10 had dramatically decreased in NMT as compared to LMT. 

Furthermore, after evaluating the insulin resistance-related genes, we found that phosphoenolpyruvate carboxykinase 1 (PCK1) expression increased considerably in NMT in comparison to LMT, while no significant difference was observed in the transcripts for glucose-6-phosphatase (*G6PC*) between the two groups. Moreover, all evaluated fibrosis-related genes, including matrix metallopeptidase-2 (*MMP*-2), *TGF-β*, and collagen type I alpha 1 chain (*COL1A1*), showed significant upregulation in the NMT.

Considering the secretome of microtissues for some key player proteins involved in NAFLD, we demonstrated that the production of all evaluated markers, i.e., IL-6, TGF-β, and P3NP (as a marker for liver fibrosis score in clinical assessment (Chen et al., 2022[9]) was considerably higher in NMT in comparison to that of LMT (Figure 3d(Fig. 3)). Accordingly, immunofluorescence staining confirmed that there are more vimentin and α-SMA positive cells in NMT (Supplementary Figure 4). 

Taken together, these results show that liver microtissue, persistently supplemented by FFAs for 8 days, could reflect steatosis-, inflammation-, and fibrosis-like pathology.

### Evaluation of NAFLD initiation and progression in NMT

**Figure 4:**
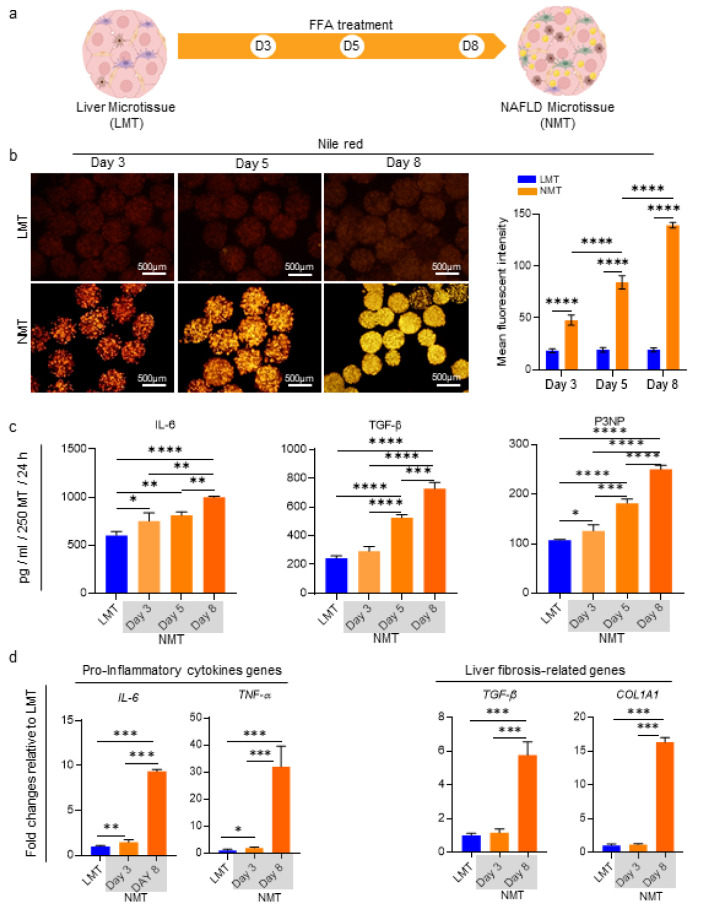
Evaluation of NAFLD initiation and progression in the FFA-treated microtissue. a) Schematic display of the study design and sampling time points. b) Representative fluorescence micrographs to visualize accumulated lipids in the microtissues, using Nile red staining on days 3, 5, and 8. Quantification of accumulated lipids in the microtissues during the NAFLD model development, using mean fluorescence intensity assessment, represented in the graph (n = 6). c) Quantification of secreted IL-6, TGF-β, and P3NP in the microtissues supernatant during the NAFLD model development, using ELISA and considering LMT on day 3 as control (n =3). d) Relative gene expressions involved in pro-inflammatory cytokines production and liver fibrosis on days 3 and 8 of NAFLD model development. Data were normalized against *GAPDH *and are presented as fold changes calibrated to expression values of the LMT on day 3 (n = 3). Data are shown as mean *± *SD. *p < 0.05, **p < 0.01, ***p < 0.001 and ****p < 0.0001.

To explore the progression of disease from steatosis to NASH and fibrosis, the NMT was assessed on days 3, 5, and 8 following FFAs supplementation (Figure 4a(Fig. 4)). Nile red staining on these days displayed a gradual increase for lipid accumulation numbers in a time-dependent manner in the NMT which were significantly higher compared to those of LMT at the same time and also in comparison to the NMT at the former time points (Figure 4b(Fig. 4)). 

Evaluating the microtissue secretome for IL-6, TGF-β, and P3NP on days 3, 5, and 8 demonstrated that except for TGF-β on day 3, the secretion of these factors was significantly higher in NMT compared to that in LMT on day 3. Furthermore, since these secretions were of a time-dependent quality, there was an increasing trend where almost in each time point secretion was considerably higher in comparison to its previous time point (Figure 4c(Fig. 4)).

Since the secretion of pro-inflammatory cytokine (IL-6) and fibrosis related proteins (TGF-β and P3NP) showed a significant increase in a time-dependent manner, which indicates the progression of the disease to NASH, the expression levels for inflammatory (e.g, *IL-6*,* TNF-α*) and fibrosis-related genes (e.g, *TGF-β*, *COL1A1*) on days 3 and 8 were compared. The significant upregulation of* IL-6* and *TNF-α* on days 3 and 8, and also* TGF-β* and *COL1A1* on day 8 indicate that NMT reflects the NASH state on day 3 as well as the progression of the disease to the fibrosis stage on day 8 (Figure 4d(Fig. 4)).

### Assessment for stability or reversibility of the NAFLD microtissue 

**Figure 5:**
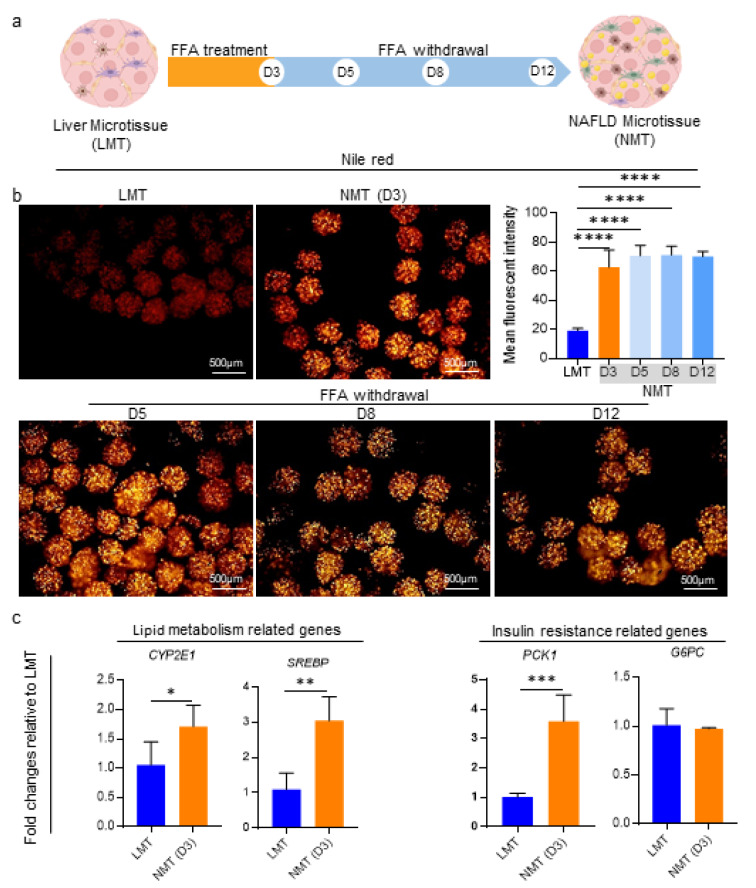
Assessment for stability or reversibility of the NAFLD microtissues. a) Schematic displaying of the experiment's design and sampling time points. As it is shown in the scheme, to evaluate the stability or reversibility of the NAFLD model, FFA supplementation was stopped on day 3. b) Representative fluorescence micrographs and quantification of mean fluorescence intensity for accumulated lipids in the microtissues, using Nile red staining. The data demonstrates that there is significantly more lipid storage in the NMT at day 3 and this condition remains constant till day 12 despite FFA withdrawal (n = 6). c) Relative gene expressions related to lipid metabolism and insulin resistance on day 3. The significant differences in the evaluated parameters confirm the NAFLD model established on day 3; therefore, this day could be considered to initiate an anti-NAFLD drug assessment (n = 3). Data are shown as mean *± *SD. **p < 0.01, ***p < 0.001 and ****p < 0.0001.

Prior to drug evaluation on NAFLD microtissue, we investigated if the NMT could maintain the disease state or would revert to a healthy condition upon stopping the FFAs' supplementation. To answer this, after the initial 3 days of FFAs treatment, the NMT culture condition was switched to FFA-free medium and continued up to day 12 (Figure 5a(Fig. 5)). Using Nile red, we found that the amount of intracellular lipids accumulated on day 3 could be maintained till day 12 even after FFAs withdrawal (Figure 5b(Fig. 5)). To investigate the cause of accumulated lipids persisting after FFAs withdrawal, the transcripts for some genes related to lipid metabolism and insulin resistance were evaluated on day 3. These results indicated that *CYP2E1*,* SREBP*, and *PCK1* genes were significantly upregulated in NMT as compared to LMT (Figure 5c(Fig. 5)). 

Considering the data collected together with the significantly higher IL-6 gene expression and cytokine production (Figure 4c and d(Fig. 4)), it could be expected that the steatosis established in NMT on day 3 and maintained up to day 12 even in the FFA-free condition. This could be explained by the inflammation and *de novo* lipogenesis process.

### The NMT drug responsiveness 

**Figure 6:**
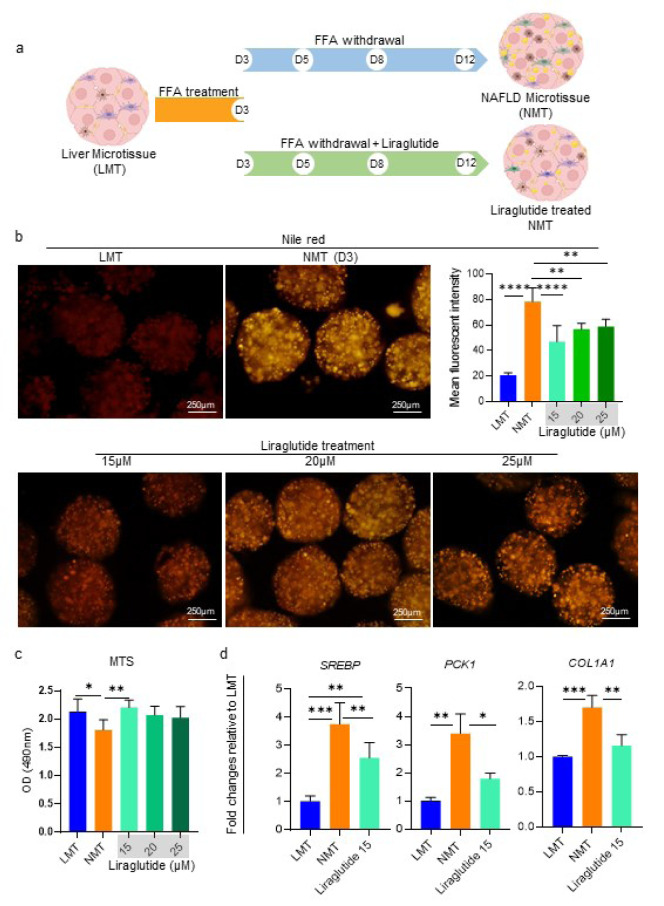
Evaluation of the NAFLD microtissue responsiveness to Liraglutide as an anti-NAFLD drug candidate. a) Schematic displaying of the experiment design and sampling time-point. After 3 days of exposure to FFA, the NMT was treated with a concentration of 15, 20, or 25 μM Liraglutide in the FFA withdrawal medium, and the sampling was done on day 12. b) Representative fluorescence micrographs and quantification of mean fluorescence intensity for accumulated lipids in the microtissues, using Nile red staining. The data demonstrates that the NMT is responsive to the drug in a dose-dependent manner, in terms of lipid accumulation, and the most effective dose of Liraglutide is 15 μM (n = 6). c) Cytocompatibility analysis for the selected doses of Liraglutide on day 12 using MTS assay (n = 6). d) Relative gene expressions on day 12 to evaluate the responsiveness of the NMT to the treatment of 15 μM Liraglutide at the transcription level. Data were normalized against *GAPDH *and are presented as fold changes calibrated to expression values of LMT (n = 3). Data are shown as mean *± *SD. *p < 0.05, **p < 0.01, ***p < 0.001 and ****p < 0.0001.

To investigate whether the generated NMT has drug responsiveness and could be used as a platform for pharmacological studies, after NAFLD establishment in the initial 3 days, the NMT was exposed to different doses (15, 20, and 25 μM) of a candidate anti-steatotic drug, Liraglutide, from day 4 to 12 (Figure 6a(Fig. 6)). Using Nile red staining, it was demonstrated that NMT has drug responsiveness capability in which dramatic lipid loss was observed in all Liraglutide treated groups on day 12 compared to NMT (Figure 6b(Fig. 6)). Evaluating cell viability, we found that none of the selected dosages of Liraglutide was toxic (Figure 6c(Fig. 6)). Based on the principle of choosing the lowest effective dose, gene expression for NMT, exposed to 15 μM Liraglutide, was evaluated. The results confirmed NMT's drug responsiveness and significant downregulations in gene expression involved in lipid metabolism (*SERBP*), insulin resistance (*PCK1*), and fibrosis (*COL1A1*) (Figure 6d(Fig. 6)).

## Discussion

Despite the global increase in the prevalence of NAFLD, no effective therapeutic medicine to target hepatic steatosis has been approved yet. Therefore, there is an urgent need to develop a reliable human *in vitro* NAFLD model that captures the disease's hallmarks, facilitates the study of the mechanism of disease, and enables the rapid evaluation of various candidate drugs in a dish. Moreover, a relevant *in vitro* liver model should be cost-effective, reproducible, and scalable in high-throughput drug discovery pipeline. During the past decades, various *in vitro* NAFLD models have been developed. Since 2D cellular platforms do not mimic the complexity of liver tissue, 3D culture systems have recently emerged as appealing tools for studying disease mechanism and investigating therapeutic interventions. On the other hand, a suitable *in vitro* human NAFLD model needs all the key player cells and the appropriate ECM to recapitulate the disease stages from initial inflammation to lipid accumulation and future fibrosis (Aasadollahei et al., 2023[1]). 

In the present study, we made an attempt to bioengineer a scalable 3D liver microtissue composed of four cell types, including Huh-7, THP-1, LX-2, and HUVEC, encapsulated in a composite hydrogel containing LEMgel. According to our previous study, regarding cell viability and microtissue functionality, the diameter of the generated microtissues were optimized at 500 μm (Darakhshan et al., 2020[13]). The uniform spherical shape of the microtissues, as an important feature, provides a linear gradient in oxygen and nutrients from peripheral to central part of the LMT similar to native hepatic lobule (Kietzmann, 2017[31]). Considering cell viability and hepatic functionality in terms of albumin secretion and urea production, we demonstrated a considerable impact of LEMgel for the generation of both LMT and NMT. In this context, Lee and colleagues have shown that the liver ECM significantly enhances the viability, functionality, and lineage-specific maturation of stem cell-derived hepatocyte-like cells (Lee et al., 2019[33]). Furthermore, our previous studies demonstrated that LEMgel- and LEMgel-derived microparticles improve liver-specific functions of the hepatic cell lines, primary hepatocytes, and hepatocyte-like cells (Heydari et al., 2021[29]; Saheli et al., 2018[44]; Zahmatkesh et al., 2021[54]). 

To produce NMT, we used FFAs treatment which is affordable and was previously demonstrated as the more appropriate approach for NAFLD induction and progression to future fibrosis (Cho et al., 2021[11]). The bioengineered NMT showed all the hallmarks of NASH (Boeckmans et al., 2018[7]) in terms of alteration in the lipid metabolism-related genes' (*CYP2E1*, *SREBP*, and *CD36*) expression, intracellular lipid accumulation (represented through Oil red and Nile red staining), inflammatory responses (detected by *TNF-α*, *IL-6*, and *IL-10* genes expression and IL-6 secretion), and fibrosis (evidenced by *MMP-2*, *TGF-β*, and *COL1A1* genes expression and TGF-β and P3NP secretion). Likewise, Ouchi and colleagues have reported that multi-cellular liver organoids differentiated from hPSCs, when exposed to FFAs, successfully recapitulate the key features of steatohepatitis, particularly inflammation, lipid accumulation, and fibrosis (Ouchi et al., 2019[41]). 

Following the NMT generation and characterization, the stability or self-reversibility of the NAFLD model, as an important point for pharmacological evaluations, was considered. The results demonstrated that the lipids accumulated in the microtissue during the initial 3 days of FFAs supplementation could be maintained irreversibly up to day 12 after FFAs withdrawal. This phenomenon may be due to the upregulation in lipid metabolism-related (*CYP2E1* and *SREBP*) and insulin resistance (*PCK1*) genes, coinciding with higher *IL-6* gene expression and cytokine production which triggers inflammatory response on day 3. These results are in contrast with Lasli and colleagues' findings. They showed that the accumulated lipids in HepG2 spheroids were alleviated to the basal level following 6 days of FFAs withdrawal. Furthermore, they found that in a hepatic spheroid containing both HepG2 and HUVEC, the recovery rate was delayed, but eventually it achieved the basal level of accumulated lipids on day 8 (Lasli et al., 2019[32]). However, in another study, they demonstrated that intracellular lipid accumulation in HepG2-based spheroid containing human Kupffer cells was not reversible (Suurmond et al., 2019[48]). The most credible aspect of an *in vitro* disease model is its capacity to reproduce the complications observed clinically. Therefore, as a progressive disease, it is critical for an *in vitro* NAFLD model not to recover autonomously.

Up to now, there has been no approved drug for NASH treatments; however, several clinical trials with various drugs are ongoing. Precise evaluations of the candidates' drugs on an appropriate *in vitro* NAFLD model recapitulating the disease can further validate their reliability. Liraglutide, as an analog of the glucagon-like peptide-1 (GLP-1), is a promising drug candidate which is being assessed in a phase II clinical trial for NASH (Armstrong et al., 2016[4]). It has been proven that Liraglutide improves hepatic insulin sensitivity and reduces liver inflammation, *de novo* lipogenesis, and hepatic steatosis (Armstrong et al., 2014[3]). 

In the current study, to assess drug responsiveness, after NAFLD's establishment, the generated NMT was exposed to Liraglutide. Although all doses of Liraglutide (15, 20, and 25 μM) used in our study diminished intracellular lipid accumulation considerably, the highest improvement occurred with the 15 μM dose. This possibly occurred due to the fact that Liraglutide contains a palmitic acid group; therefore, applying high doses of Liraglutide means an increase in FFAs in the culture medium and its accumulation in the cells. Moreover, at the level of gene expression, improving insulin sensitivity, anti-inflammatory, and anti-fibrotic effects of Liraglutide were evident in the treated NMTs.

Interestingly, our results are consistent with a study currently in phase II clinical trial, in which Liraglutide was administered for 72 weeks and resulted in the resolution of fibrosis among NASH patients with stage 1 liver fibrosis (Newsome et al., 2021[38]). Furthermore, Pingitore and colleagues demonstrated that Liraglutide prevents the development of steatosis and COL1A1 level in an *in vitro *NAFLD model generated as 3D spheroids composed of HepG2 and LX-2 cell lines (Pingitore et al., 2019[42]).

One of the critical issues regarding human *in vitro* modeling is the source of human cells in terms of availability, affordability, reproducibility, and scalability. Human primary cells are faced with obstacles in accessibility, donor-dependent variability, and *in vitro* instability, that limit the generation of reproducible and scalable *in vitro* NAFLD models for high-throughput evaluations (Zeilinger et al., 2016[56]). Stem cells hPSCs are another unlimited cell source for *in vitro* modeling. However, there is no global definitive protocol for the mass production of fully mature differentiated cells to be functionally equivalent to the primary cells. Therefore, the limited reproducibility and reliability for the very expensive stem cell-derived differentiated cells and their resultant *in vitro* models are critical bottle necks to making them commercially applicable. 

Immortalized cell lines, as the third source for human cells, are widely available with unlimited proliferation capacity. These cell lines are cost-effective and reproducible, the qualities which make them appropriate for *in vitro* high-throughput applications. However, a major drawback of these cell lines is their abnormal phenotype, altered metabolism, and immaturity (Beckwitt et al., 2018[6]). Therefore, there is a general tendency to rely on human primary cells or those differentiated from stem cells to create human models *in vitro*. However, our previous studies have proven that human cell lines, particularly Huh-7 as a substitute for hepatocyte, can still be effective when provided with a suitable role, in terms of co-culturing them with non-parenchymal cells and supporting them with liver-specific ECM. In such an appropriate microenvironment, the resultant liver organoid, liver microtissue or hepatic patch demonstrated considerable enhancements in liver-specific functions, particularly in relation to toxicological response and drug sensitivity (Darakhshan et al., 2020[13]; Nobakht Lahrood et al., 2020[39]; Saheli et al., 2018[44]). 

Overall, in the present study, we have successfully developed a cost-effective and scalable liver microtissue that can partially mimic the development and progression of NAFLD through treatment with FFAs. Furthermore, the stability and irreversibility of the NAFLD microtissue, as well as its responsiveness to drug treatment, which are crucial factors for pharmacological applications, were approved. Despite its numerous advantages, particularly the ability to lipids accumulation, the microtissue lacks metabolic zonation and fails to reproduce important features of the disease, such as lipogranulomas, fibrotic streaks, and ductular reaction. 

To date, all available alternative methods used to study human NAFLD, whether laboratory animals or *ex vivo* and *in vitro* models, have their own limitations. As a result, there is no single model that accurately reflects the human condition and effectively demonstrates the development, progression, underlying mechanisms, and treatment of NAFLD (Dichamp et al., 2023[15]; Ghallab et al., 2021[23]; Godoy et al., 2016[26]; Green et al., 2018[28]). However, there is hope on the horizon with the emergence of new technologies such as liver organoid generation, liver organoid-on-a-chip, whole body-on-a-chip, gene regulatory network analyses, and systems biology. These advancements will contribute to the development of more accurate and relevant *in vitro* models for studying NAFLD.

## Conclusion

In the current study, we developed a biomimetic, cost-benefit, and reproducible 3D *in vitro* liver microtissue which consists of Huh-7, THP-1, and LX-2 cell lines and HUVEC embedded in a composite containing liver extracellular matrix and alginate. The bioengineered scalable microtissues successfully captured some of the major NAFLD features following FFAs supplementation. Therefore, they could be utilized to study the cellular and molecular mechanism of the disease initiation, progression or prevention. Moreover, their potential to response to Liraglutide demonstrated the microtissue applicability in drug screening in the different stages of NAFLD. However, novel technologies, such as 3D bioprinting and on-a-chip platform, could be implemented to improve this model for biomedical applications and commercialization.

## Notes

Massoud Vosough and Abbas Piryaei (Department of Biology and Anatomical Sciences, School of Medicine, Shahid Beheshti University of Medical Sciences, Tehran, Iran; Tel: +989126971784, E-mail: piryae@sbmu.ac.ir) contributed equally as corresponding author.

## Declaration

### Conflict of interest

The authors declare that there is no conflict of interest. 

### Acknowledgments

We would like to express our sincere gratitude to Dr. Zahra Farzaneh, Dr. Mona Saheli, Dr. Sara Darakhshan, Forough Azam Sayyahpour, Hassan Ansari, and Mahmoud Mahmoudi Asl for their technical supports.

### Funding

This project was financially supported by grants from Royan Institute (Grant No 99000166), Shahid Beheshti University of Medical Sciences (Grant No 27788), and Iran National Science Foundation (INSF, Grant No 4014930), Tehran, Iran. 

### Author contributions

NA performed the main experiments, data collection and analyses, and drafted the manuscript. MAH, MAC, and MA contributed to the development of the self-built air-driven droplet generator system for the mass production of microtissues and histological examinations. SAZ, MV and AP conducted the data management and interpretation. MV and AP conceptualized the hypothesis, revised the manuscript draft critically, and provided resources. All authors have read and approved the final manuscript.

## Supplementary Material

Supplementary information

## Figures and Tables

**Figure 1:**
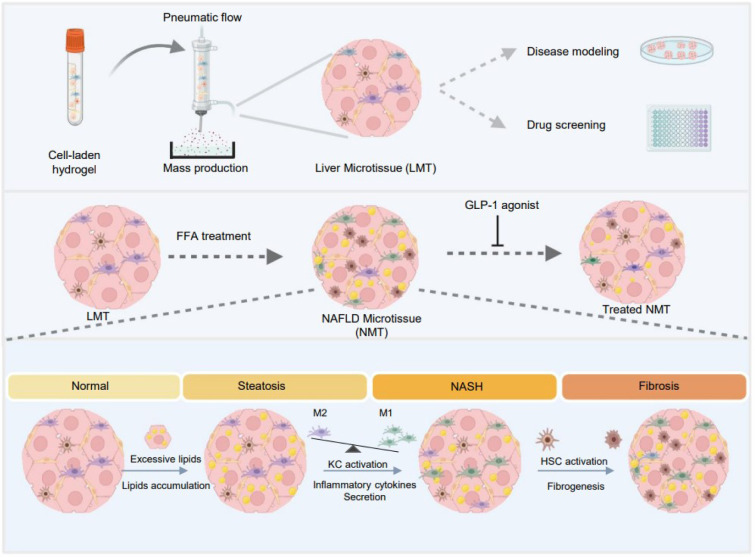
Graphical abstract
