# Supplementary material for: Bioengineering scalable and drug-responsive in vitro human multicellular non-alcoholic fatty liver disease microtissues encapsulated in the liver extracellular matrix-derived hydrogel
Source: EXCLI J. 2024 Mar 25;23:421–40. doi: 10.17179/excli2023-6878 (PMC11089098; doi:10.17179/excli2023-6878)
Supplement: Supplementary information [file EXCLI-23-421-s-001.pdf]

EXCLI Journal 2024;23:421-440 – ISSN 1611-2156
Received: December 12, 2023, accepted: March 06, 2024, published: March 25, 2024

Supplementary information to:

Original article:

BIOENGINEERING SCALABLE AND DRUG-RESPONSIVE *IN VITRO* HUMAN MULTICELLULAR NON-ALCOHOLIC FATTY LIVER DISEASE MICROTISSUES ENCAPSULATED IN THE LIVER EXTRACELLULAR MATRIX-DERIVED HYDROGEL

Negar Asadollahi[1,8], Mohammad Amin Hajari[2], Mahmoud Alipour Choshali[3], Mohammad Ajoudanian[4], Seyed Ali Ziai[5], Massoud Vosough[1,6]*, Abbas Piryaei[7]*

1 Department of Regenerative Medicine, Cell Science Research Center, Royan Institute for Stem Cell Biology and Technology, ACECR, Tehran, Iran
2 Department of Cell Engineering, Cell Science Research Center, Royan Institute for Stem Cell Biology and Technology, ACECR, Tehran, Iran
3 Department of Stem Cells and Developmental Biology, Cell Science Research Center, Royan Institute for Stem Cell Biology and Technology, ACECR, Tehran, Iran
4 Department of Medical Biotechnology, School of Advanced Technologies in Medicine, Shahid Beheshti University of Medical Sciences, Tehran, Iran
5 Department of Pharmacology, School of Medicine, Shahid Beheshti University of Medical Sciences, Tehran, Iran
6 Experimental Cancer Medicine, Institution for Laboratory Medicine, Karolinska Institute, Huddinge, Sweden
7 Department of Biology and Anatomical Sciences, School of Medicine, Shahid Beheshti University of Medical Sciences, Tehran, Iran
8 Department of Developmental Biology, University of Science and Culture, Tehran, Iran

* **Corresponding authors:** Abbas Piryaei, Department of Biology and Anatomical Sciences, School of Medicine, Shahid Beheshti University of Medical Sciences, Tehran, Iran.
Tel: +989126971784, E-mail: piryae@sbmu.ac.ir
Massoud Vosough, Department of Regenerative Medicine, Cell Science Research Center, Royan Institute for Stem Cell Biology and Technology, ACECR, Tehran, Iran.
Tel: +989121196454, E-mail: masvos@royaninstitute.org

https://dx.doi.org/10.17179/excli2023-6878

This is an Open Access article distributed under the terms of the Creative Commons Attribution License (http://creativecommons.org/licenses/by/4.0/).

EXCLI Journal 2024;23:421-440 – ISSN 1611-2156
Received: December 12, 2023, accepted: March 06, 2024, published: March 25, 2024

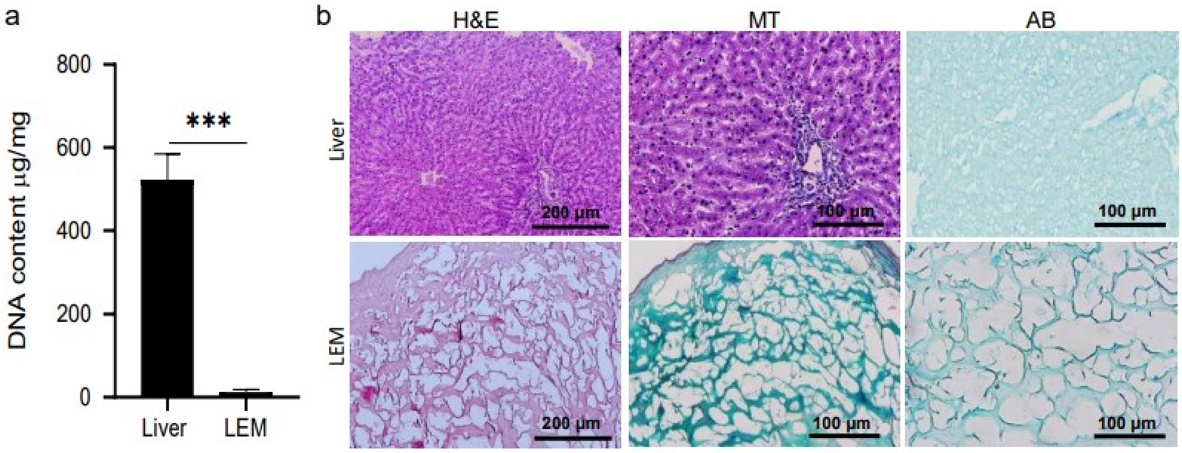

Supplementary Figure 1: Characterization of the decellularized liver-derived extracellular matrix (LEM). **a)** Quantification of DNA content in the liver and LEM. **b)** Histological evaluations of the liver and the resultant LEM. Hematoxylin and eosin (H&E) staining showed that, compared to the native liver, there are no nuclei in the LEM. Preservation of collagen and glycosaminoglycans (GAGs) in the LEM was demonstrated using Masson's trichrome (MT) and Alcian blue (AB) staining, respectively.

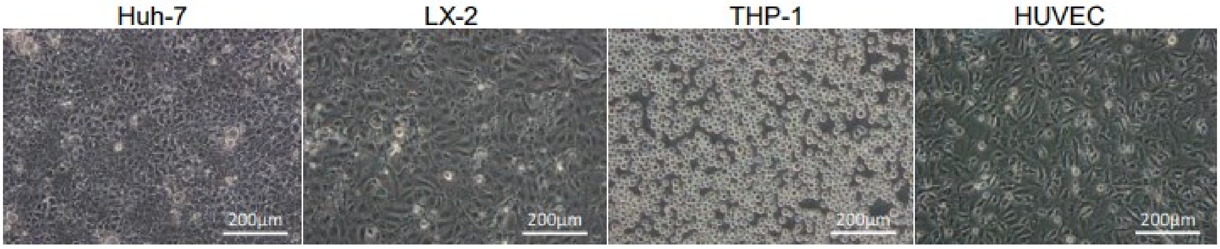

Supplementary Figure 2: Phase-contrast micrographs of Huh7, LX-2, and THP-1 cell lines and HUVEC, demonstrated the cells morphology in two-dimensional culture condition.

EXCLI Journal 2024;23:421-440 – ISSN 1611-2156
Received: December 12, 2023, accepted: March 06, 2024, published: March 25, 2024

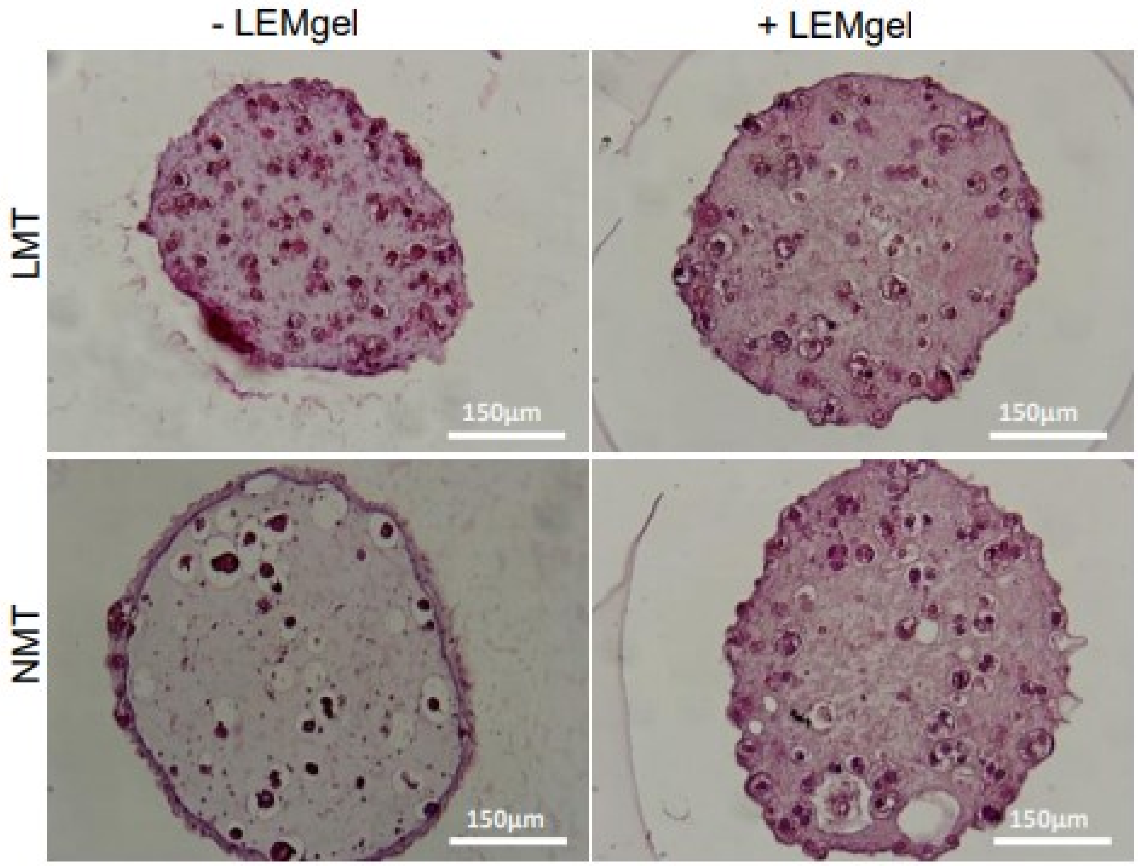

Supplementary Figure 3: Light micrographs from Hematoxylin and eosin-stained sections of the LMTs and NMTs generated in the presence or absence of LEMgel on day 8. As it is visible, the overall structures of the +LEMgel microtissues are more appropriate with homogenous distribution of live cells. While the –LEMgel microtissues revealed visible destruction in their structure and there are many necrotic cells with pyknotic nuclei, particularly in the NMT.

EXCLI Journal 2024;23:421-440 – ISSN 1611-2156
Received: December 12, 2023, accepted: March 06, 2024, published: March 25, 2024

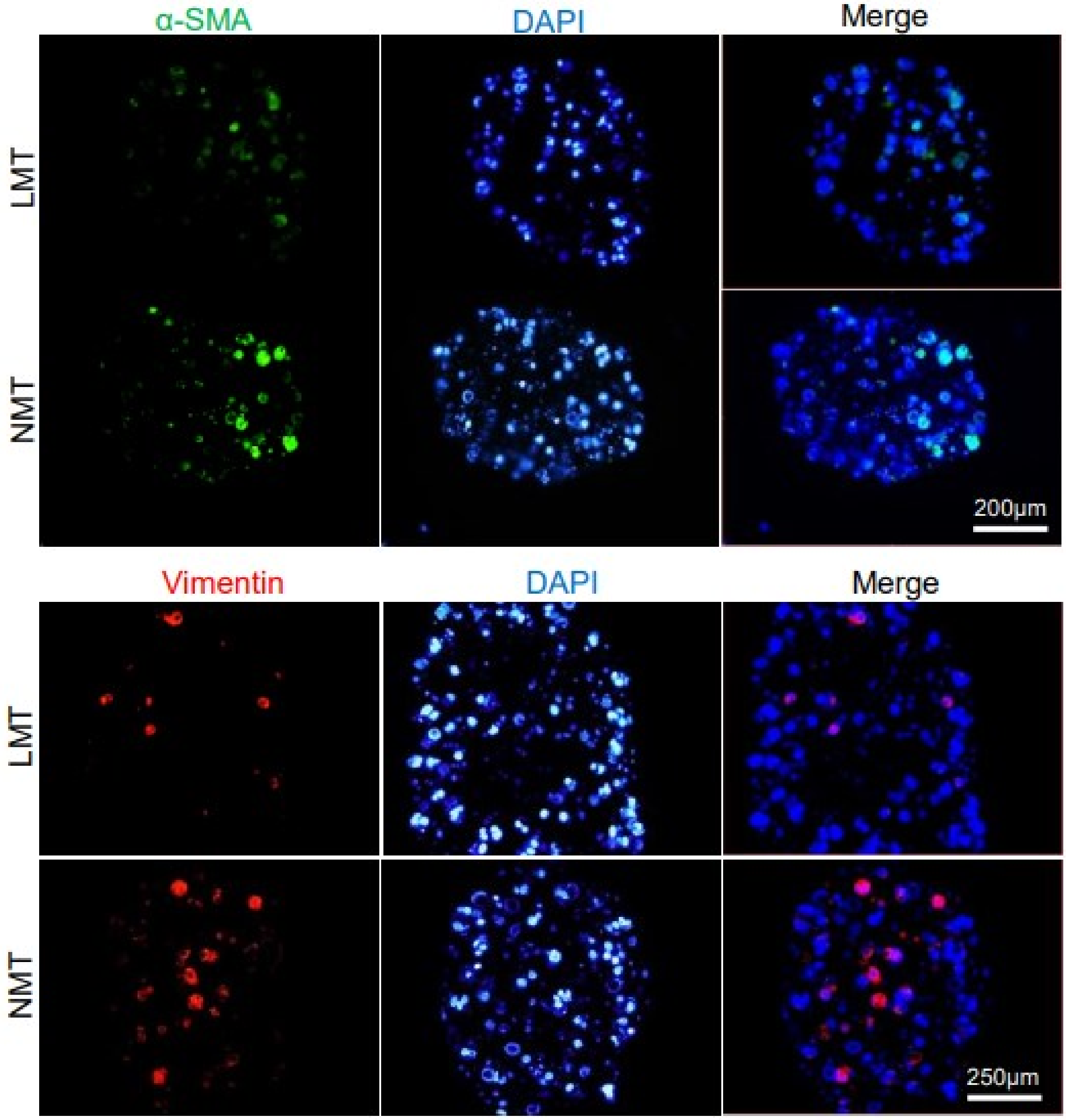

Supplementary Figure 4: Representative immunofluorescence micrographs from the microtissue sections stained against Vimentin and α-SMA on day 8. The micrographs revealed that there are more Vimentin-positive and α-SMA-positive cells in the NMTs in comparison to the LMTs.

EXCLI Journal 2024;23:421-440 – ISSN 1611-2156
Received: December 12, 2023, accepted: March 06, 2024, published: March 25, 2024

Supplementary Table 1: Primer sequences used in qRT-PCR

Genes	Sequence
CPT1	F: ATCAATCGGACTCTGGAAACGG<br>R: TCAGGGAGTAGCGCATGGT
CD36	F: CTTTGGCTTAATGAGACTGGGAC<br>R: GCAACAAACATCACCACACCA
SREBP	F: CGGAACCATCTTGGCAACAGT<br>R: CGCTTCTCAATGGCGTTGT
CYP2E1	F: GTGATGCACGGCTACAAGG<br>R: GGGTGGTCAGGGAAAACCG
IL 6	F: AGGAGACTTGCCTGGTGAAA<br>R: CAGGGGTGGTTATTGCATCT
IL 8	F: TAGCAAAATTGAGGCCAAGG<br>R: AGCAGACTAGGGTTGCCAGA
IL 10	F: AAGCTGAGAACCAAGACCCA<br>R: AAGGCATTCTTCACCTGCTC
TGF-β	F: GAAACCCACAACGAAATCTATGA<br>R: TAACTTGAGCCTCAGCAGAC
TNF-α	F: CCTCTCTCTAATCAGCCCTCTG<br>R: GAGGACCTGGGAGTAGATGAG
MMP-2	F: ATTGTATTTGATGGCATCGCTC<br>R: ATTCATTCCCTGCAAAGAACAC
COL1A1	F: ATGCCTGGTGAACGTGGT<br>R: AGGAGAGCCATCAGCACCT
PCK1	F: GGCTGAAGAAGTATGACAACTG<br>R: AAATCCTCCTCTGACATCCA
G6P	F: AACATCGCCTGCGTTATCCTC<br>R: ACGTCCCGGATGATCCCAA
GAPDH	F: GAAATCCCATCACCATCTTCC<br>R: GGCTGTTGTCATACTTCTCAT
